# Coupling of cell growth modulation to asymmetric division and cell cycle regulation in *Caulobacter crescentus*

**DOI:** 10.1073/pnas.2406397121

**Published:** 2024-10-03

**Authors:** Skye Glenn, Alessio Fragasso, Wei-Hsiang Lin, Alexandros Papagiannakis, Setsu Kato, Christine Jacobs-Wagner

**Affiliations:** ^a^Department of Biology, Stanford University, Stanford, CA 94305; ^b^Sarafan Chemistry, Engineering, and Medicine for Human Health Institute, Stanford University, Stanford, CA 94305; ^c^HHMI, Stanford University, Stanford, CA 94305; ^d^Department of Molecular, Cellular, and Developmental Biology, Yale University, New Haven, CT 06511; ^e^Department of Microbiology and Immunology, Stanford University School of Medicine, Stanford, CA 94305

**Keywords:** *Caulobacter crescentus*, cell growth, asymmetric division, cell cycle

## Abstract

In proliferating bacteria, growth rate is often assumed to be similar between daughter cells. However, most of our knowledge of cell growth derives from studies on symmetrically dividing bacteria. In many α-proteobacteria, asymmetric division is a normal part of the life cycle, with each division producing daughter cells with different sizes and fates. Here, we demonstrate that the functionally distinct swarmer and stalked daughter cells produced by the model α-proteobacterium *Caulobacter crescentus* can have different average growth rates under nutrient-replete conditions despite sharing an identical genome and environment. The discrepancy in growth rate is due to a growth slowdown associated with the cell cycle stage preceding DNA replication (the G1 phase), which initiates in the late predivisional mother cell before daughter cell separation. Both progenies experience a G1-associated growth slowdown, but the effect is more severe in swarmer cells because they have a longer G1 phase. Activity of SpoT, which produces the (p)ppGpp alarmone and extends the G1 phase, accentuates the cell cycle-dependent growth slowdown. Collectively, our data identify a coupling between cell growth, the G1 phase, and asymmetric division that *C. crescentus* may exploit for environmental adaptation through SpoT activity. This coupling differentially modulates the growth rate of functionally distinct daughter cells, thereby altering the relative abundance of ecologically important G1-specific traits within the population.

## Significance

Bacterial growth rate modulation is generally associated with changes in genetic make-up or environmental condition. This study demonstrates that the rate of cell growth can also vary between daughter cells and across cell cycle stages when nutrients are available. This is illustrated by the asymmetrically dividing α-proteobacterium *Caulobacter crescentus*, which produces two functionally distinct daughter cells that differ in average growth rate. This growth rate difference arises from a growth slowdown associated with a cell cycle phase that is longer in the motile daughter cell. Altogether, this study showcases the coupling of cell growth modulation to asymmetric division and cell cycle regulation, which may have implications for other α-proteobacteria given their cell cycle similarities to *C. crescentus*.

Cells must grow between divisions to maintain their size across generations. In bacteria, cell growth is typically assumed to be similar between daughter cells and largely constant throughout the cell cycle once normalized for cell size. However, it is unclear whether this assumption applies across bacteria, particularly asymmetrically dividing bacteria, which tend to exhibit a higher degree of cell cycle control not seen in symmetrically dividing bacteria. Asymmetric divisions and the generation of daughter cells of different sizes and fates are common among α-proteobacteria, a large class of diverse organisms that includes free-living and host-associated species (1–6). These bacteria tightly regulate their cell cycles to couple important cellular traits—like dispersal, foraging, adhesion, surface sensing, virulence, or symbiosis—to specific cell cycle phases (7–13).

**Fig. 1.**
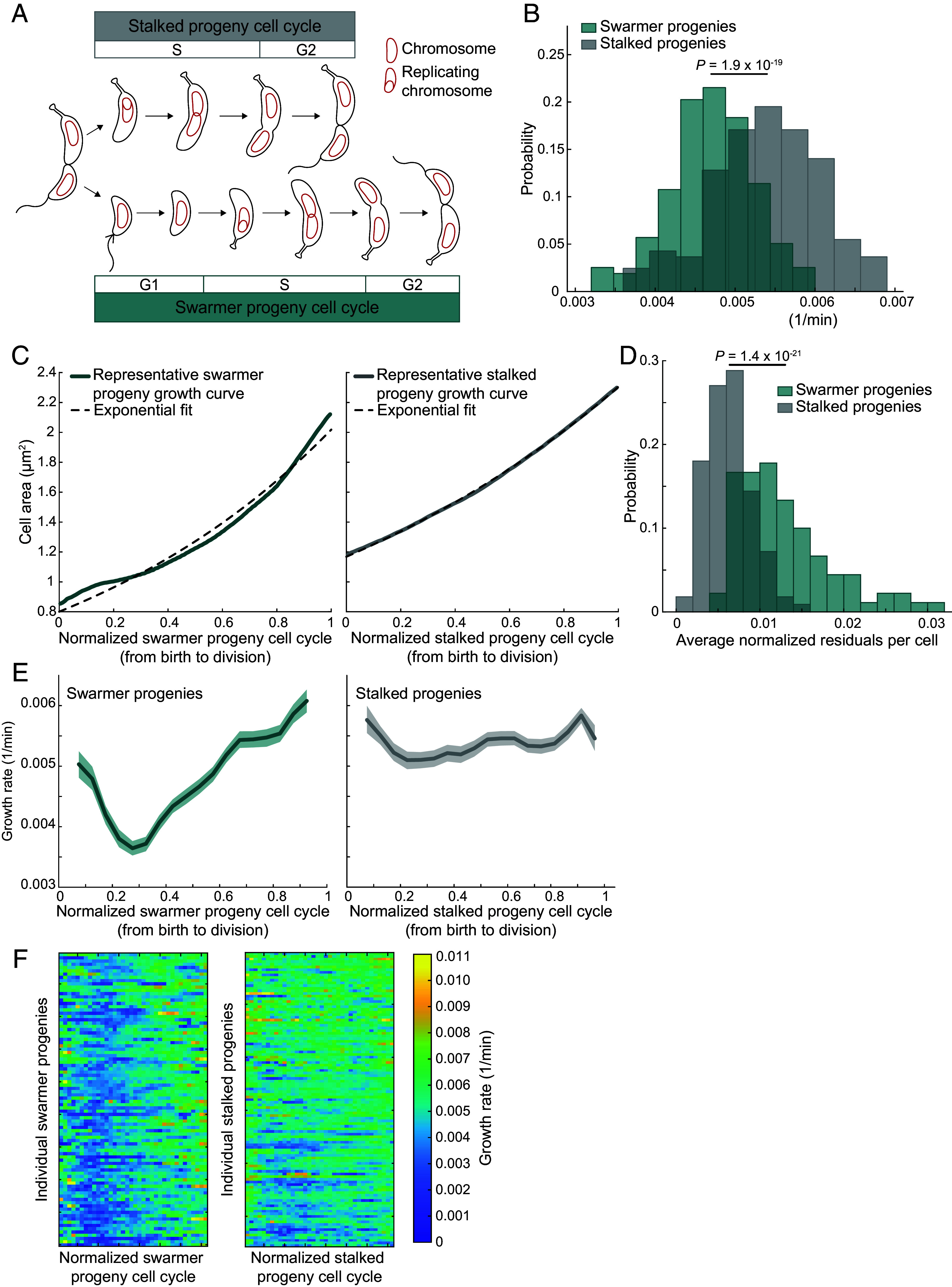
Swarmer and stalked progenies grow at different rates on average. (*A*) Prevailing view of the cell cycles of *C. crescentus* swarmer and stalked progenies. The DNA replication cycle is represented by the G1/S/G2 convention. (*B*) Distributions of average growth rate for wild-type (CB15N) swarmer (n = 158) and stalked (n = 164) progenies growing on low-agarose pads. The *P*-value was obtained using the Mann–Whitney *U* test. (*C*) Representative growth curves of a single swarmer and stalked progeny. The dotted lines are the best exponential fits for each trajectory. (*D*) Plot showing the average of the residuals to exponential fits for each cell, normalized for cell area, for the 90 swarmer progenies and 111 stalked progenies that were tracked throughout their cell cycles. *P*-value is the result from the Mann–Whitney *U* test comparing the two distributions. (*E*) Plot showing the growth rate (absolute growth rate normalized for cell area) of all tracked swarmer and stalked progenies, aligned by cell cycle unit. Solid lines and shaded areas denote mean and 95% CI of the mean from bootstrapping, respectively. (*F*) Tempograms of the growth rate for all tracked swarmer and stalked progenies sorted from shortest (*Top*) to longest (*Bottom*) interdivision time.

Asymmetric division and cell cycle controls have been extensively studied in *Caulobacter crescentus*, an α-proteobacterium naturally found in both aquatic and soil environments (14, 15). The life cycle of this organism is characterized by a developmental program that is coupled to specific cell cycle events through a sophisticated array of molecular control mechanisms and checkpoints (16, 17). These cell cycle complexities, combined with the absence of overlapping DNA replication cycles, have earned *C. crescentus* a reputation of being “eukaryote-like,” so much so that the *C. crescentus* cell cycle is typically described with the eukaryotic G1/S/G2 phase convention rather than the common bacterial B/C/D period terminology to refer to the stages before, during, and after chromosome replication (Fig. 1*A*). At each cell cycle, *C. crescentus* divides asymmetrically to produce two daughter cells of different sizes and functionalities: the smaller swarmer cell and the larger stalked cell (Fig. 1*A*) (12, 14). The stalked progeny has a thin polar appendage (stalk) with a sticky holdfast that allows the cell to colonize surfaces (14). This daughter cell is typically described as DNA replication-competent at birth, as it is thought to initiate a new round of chromosome replication immediately after cell division. Meanwhile, the swarmer daughter cell experiences a G1 phase (B period) in which it is unable to replicate its genome (Fig. 1*A*), akin to the DNA presynthetic gap of the eukaryotic cell cycle. During this G1 phase, the swarmer cell is endowed with specific cellular functionalities such as motility and surface sensing mediated by the flagellum and pili at a specific cell pole. Following the G1 phase, the swarmer cell differentiates into a stalked cell through pilus retraction, flagellum ejection, and stalk growth (the swarmer-to-stalked cell transition), and concurrently becomes competent for DNA replication (the G1/S phase transition) (Fig. 1*A*).

The cellular and molecular events that control and couple the cell cycle and development in *C. crescentus* have been dissected in significant detail (17–19). However, there remains a lack of clarity around cell growth over the complete life cycle of this organism. In *C. crescentus*, technical limitations have largely confined the quantitative study of growth to the stalked progeny (20–26). For this progeny, growth between birth and division is well described by a single exponential fit (22, 24, 25), indicating that the growth rate is relatively constant between birth and division when normalized by cell size. It should be noted that recent growth analysis of *C. crescentus* stalked progenies and *Escherichia coli* cells has brought some nuance to this view, suggesting a slight superexponential growth behavior in both organisms (27). Regardless of this subtlety, quantitative information about the growth of the *C. crescentus* swarmer progeny during its cell cycle is lacking. It is possible that the G1 phase corresponds to a growth-arrested stage, with cell growth resuming only after the G1/S phase transition when the swarmer cell differentiates into a stalked cell. Alternatively, swarmer cells may grow at the same rate (normalized by cell size) as their stalked siblings. A state of growth is more consistent with electron micrograph measurements of synchronized cell cultures suggesting that cell volume increases during the swarmer cell phase (5). However, no growth rate measurements could be extracted by this approach.

Modulation of growth rate during the cell cycle or in response to asymmetric division is known to occur in eukaryotic cells (28–31). Therefore, we set out to determine whether *C. crescentus*, an asymmetrically dividing bacterium with functional traits coupled to cell cycle progression, also modulates cell growth during its life cycle.

## Results

### Swarmer Progenies Display Lower Average Growth Rate than Stalked Progenies.

Characterizing cell growth over the complete asymmetric life cycle of *C. crescentus* has been hampered by technical limitations, as noted in the Introduction. Microfluidic devices based on cell immobilization via the holdfast have been effective at tracking the growth of stalked progenies, but not swarmer progenies (20, 22, 24, 26). While swarmer cells can be isolated for study using a synchronization technique (32), this method is not well suited for cell growth measurements as the protocol involves growth arrest through cold shock and starvation. Furthermore, the isolated cells include all swarmer cells—newborns, those about to differentiate into stalked cells, and everything in between—averaging any effect that might occur between the beginning and the end of the G1 phase. Therefore, we turned to timelapse microscopy on low-agarose (0.3%) pads containing peptone-yeast extract (PYE) medium at 30 °C, which allowed the tracking of both swarmer and stalked progenies from their moment of birth (33). Compared to more conventional 1% agarose pads, the more aqueous environment of low-agarose pads allows swarmer cells to separate from their mothers at division and swim away from their stalked siblings before occasionally reattaching to the low-agarose pad nearby (Movies S1 and S2). This cell separation event provides a clear visual indicator of the end of cell division (*Materials and Methods*).

From the collected images, we measured the cell areas of swarmer and stalked progenies at birth (*A_b_*) and division (*A_d_*), defining birth as the frame before the appearance of visible cell separation and division as the frame before the subsequent cell separation. We define the progeny cell cycle as the entirety of the cell cycle between birth and division (Fig. 1*A*). From these measurements, we calculated the average growth rate as $\frac{\ln(A_{d}/A_{b})}{\Delta t}$ where Δ*t* is the interdivision time. We found that swarmer progenies grew, on average, about 13% slower (Mann–Whitney *U* test, *P* = 1.93 × 10^−19^) compared to their stalked siblings (Fig. 1*B*). Estimating cell volume for cell size measurements yielded similar results as cell area (*SI Appendix*, Fig. S1). Therefore, we decided to use cell area to calculate growth rate as this metric was directly measured from the two-dimensional microscopy images.

### The Lower Overall Growth Rate of Swarmer Progenies Is Caused by a Transient Growth Rate Slowdown during the Cell Cycle.

To examine growth rate dynamics throughout the swarmer and stalked cell cycles, we extracted the area of cells for all frames (1.5 min imaging interval) between birth and division. As expected (21, 22, 24–26, 33), the increase in cell area of stalked progenies was well described by a single exponential fit, illustrated by a representative trace in Fig. 1 *C*, *Right*. In contrast, an exponential fit systematically failed to describe the entirety of individual swarmer progeny growth curves, also exemplified in Fig. 1 *C*, *Left*. This deviation resulted in larger fitting residuals for the tracked swarmer progenies compared to stalked progenies (Fig. 1*D*).

To assess potential systematic changes in growth along single cell cycles, we determined the growth rate of individual cells by calculating the cell area difference between time frames, normalizing for cell area, and plotting it as a function of the normalized cell cycle time from birth (zero) to division (*Materials and Methods*). We found strikingly different results between daughter cells. For stalked progenies, the variability in growth rate during the cell cycle was relatively mild (Fig. 1 *E*, *Right*). In comparison, the growth rate of swarmer progenies slowed considerably in the first third of their cell cycle, then increased to eventually reach an average growth rate close to that of the stalked progenies (Fig. 1 *E*, *Left*). At the single-cell level, there was substantial heterogeneity (Fig. 1*F*) as depicted by “tempograms,” in which each row represents the growth rate by a color scale along a single cell cycle (34). Despite this heterogeneity, the tempogram of swarmer progenies (Fig. 1 *F*, *Left*) showed evidence of slower growth rate (shown by a distinctive dark blue trough) in the first half of the cell cycles that was less apparent in the stalked progenies tempogram (Fig. 1 *F*, *Right*).

### The Growth Rate Slowdown Is Associated with the G1 Phase Irrespective of Daughter Cell Identity.

**Fig. 2.**
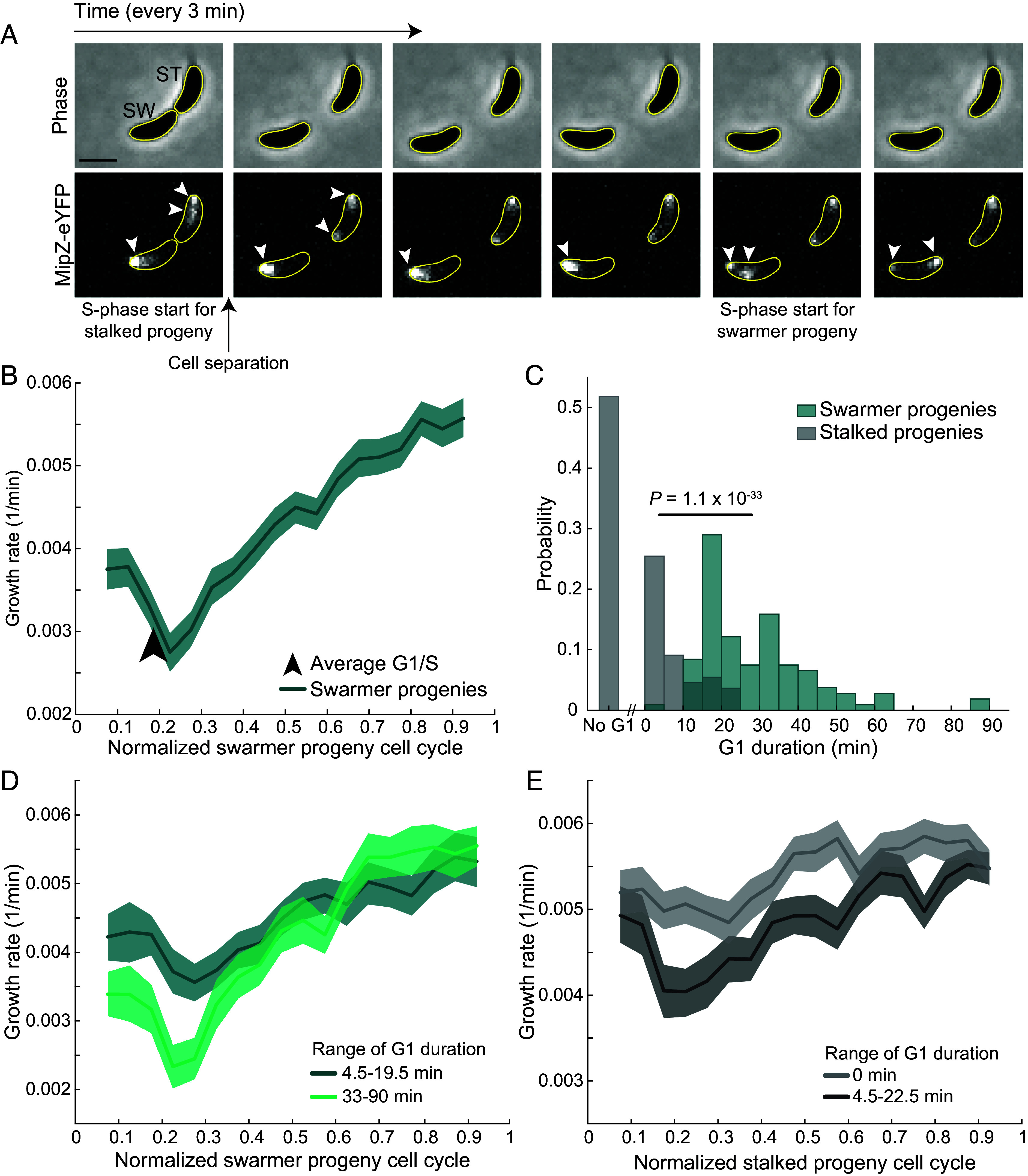
The period of slow growth is associated with time spent in the G1 phase. (*A*) Example montage of a stalked (ST) and swarmer (SW) progeny with the replication marker MipZ labeled with eYFP (strain CJW2022). Cell birth is defined as the last frame before the daughter cells are clearly separated. White arrowheads point to fluorescent MipZ-eYFP spots. Scale bar represents 1 µm. (*B*) Plot showing the growth rate of all tracked swarmer progenies (n = 107) expressing a MipZ-eYFP fusion (strain CJW2022). The solid line and shaded green area denote mean growth rate and 95% CI of the mean from bootstrapping, respectively. The black arrow represents the average G1 duration of the population as measured by MipZ-eYFP segregation. (*C*) Histogram of the absolute duration of the G1 phase across all analyzed 107 swarmer and 110 stalked progenies. *P*-value is the result from the Mann–Whitney *U* test comparing the two distributions. (*D*) Plot showing growth rate of swarmer progenies binned by the time spent in the G1 phase, which was either 4.5 to 19.5 min (n = 41 cells) or 33 to 90 min (n = 36 cells). The solid line and shaded area denote mean and 95% CI of the mean from bootstrapping, respectively. (*E*) Same as panel (*D*) but for stalked progenies with either no G1 phase (n = 57 cells) or a G1 phase between 4.5 and 22.5 min (n = 30 cells).

A major cell cycle difference between the two daughter cells of *C. crescentus* regards the G1 phase. This phase is thought to be virtually nonexistent in stalked progenies while swarmer progenies go through an extended G1 phase before they can initiate DNA replication (Fig. 1*A*). In PYE medium, the swarmer G1 phase has been estimated to last 20 to 33% of the cell cycle (24, 35, 36), which is within the same range as the observed growth rate decrease in the swarmer cell cycle (Fig. 1 *E*, *Left*). As such, we hypothesized that the growth rate change in swarmer progenies is associated with the G1 phase duration. To observe DNA replication in the context of the growth rate change, we performed low-agarose timelapse microscopy on a strain that contains MipZ-eYFP, a translational fusion of a yellow fluorescent protein variant (eYFP) to the DNA replication marker MipZ (Fig. 2*A* and Movie S3). Fluorescent patches of MipZ-eYFP track the segregation of chromosomal origins of replication (37), which occurs shortly after DNA replication initiation (38). Here, we defined the G1 phase as the time between cell birth and the appearance of two MipZ-eYFP patches in the cell (Fig. 2*A* and Movie S3).

We found that, under our experimental conditions, the average duration of the G1 phase in swarmer progenies approximately corresponded to the first 20% of the cell cycle (~0.2 cell cycle units), slightly preceding the reversal of growth rate from slowing to accelerating (Fig. 2*B*). Analysis of individual cells revealed a high degree of variability in G1 duration across swarmer progenies, ranging between 4.5 and 90 min (Fig. 2*C*). When we binned swarmer progenies by the duration of time spent in the G1 phase, we found that cells with longer G1 phases had a more pronounced slowing of growth in the early stages of the cell cycle (Fig. 2*D*).

We also noticed that a portion of the stalked progeny population experienced a short G1 phase (Fig. 2*C*). By comparing the growth rate of stalked progenies with and without a G1 phase, we found that those with a G1 phase (of at least 4.5 min for comparison with swarmer progenies) also exhibited a slight reduction in growth rate in the early stages of the cell cycle compared to those with no G1 phase (Fig. 2*E*). This suggests that slow growth early in the cell cycle is not a phenomenon specific to cells with swarmer identity, but rather a more general feature of experiencing a phase without DNA replication.

### Stalked Progenies Display a G1-Associated Growth Slowdown That Starts in the Mother Cells before Cell Separation.

By convention, the G1 phase is defined to start at cell birth, i.e., at the completion of cell division when the two daughter cells physically separate. Our low-agarose timelapse technique, which clearly identifies the time of daughter cell separation with the swarmer progeny moving away from its sibling (Movies S1 and S2), revealed that most stalked progenies had no G1 phase by this definition (Fig. 2*C*). This implies that, in these cells, DNA replication initiated in the mother cell before daughter cell separation. This is likely because, in *C. crescentus*, the timing of membrane invagination is uncoupled during cell division, with the inner membrane fusing well before (18 ± 5 min) the outer membrane (39). This uncoupling between inner and outer membranes results in the generation of late predivisional cells with two functionally separate cytoplasmic compartments. In fact, completion of cytokinesis (i.e., when one cytoplasm is divided into two) in the late predivisional cell triggers the differential activation of the cell cycle regulators CtrA and DivK between the cytoplasmic compartments of the future progenies (10, 39, 40). Therefore, we reasoned that the true start of the G1 phase likely corresponds to the end of cytokinesis in the mother cell rather than cell separation.

To examine the possibility that stalked cells may also experience a G1 phase and an associated growth rate slowdown prior to cell separation, we took advantage of the machine learning-based Omnipose/SuperSegger software (41, 42) and a cell segmentation model that we recently trained on phase contrast images of *E. coli* cells (43). To benchmark this cell segmentation model on *C. crescentus*, we used images of *C. crescentus* cells carrying the cell cycle response regulator DivK fused to a fluorescent protein. DivK changes its localization pattern from a bipolar to unipolar accumulation in response to completion of cytokinesis in the late predivisional cell (*SI Appendix*, Fig. S2*A*) (10). We found that our cell segmentation model performed well at splitting cells that had undergone cytokinesis but not yet daughter cell separation, as all (46 out of 46) analyzed late predivisional cells with unipolar localization of fluorescently labeled DivK were split into two cells (*SI Appendix*, Fig. S2 *B* and *C*). Furthermore, a large majority of predivisional cells (85 out 105) with bipolar DivK localization (i.e., prior to completion of cytokinesis) remained segmented as single cells (*SI Appendix*, Fig. S2 *B* and *C*). These results indicate that, while not perfect, the model adequately split most cells near the time of cytokinesis completion based on phase contrast cell features alone.

**Fig. 3.**
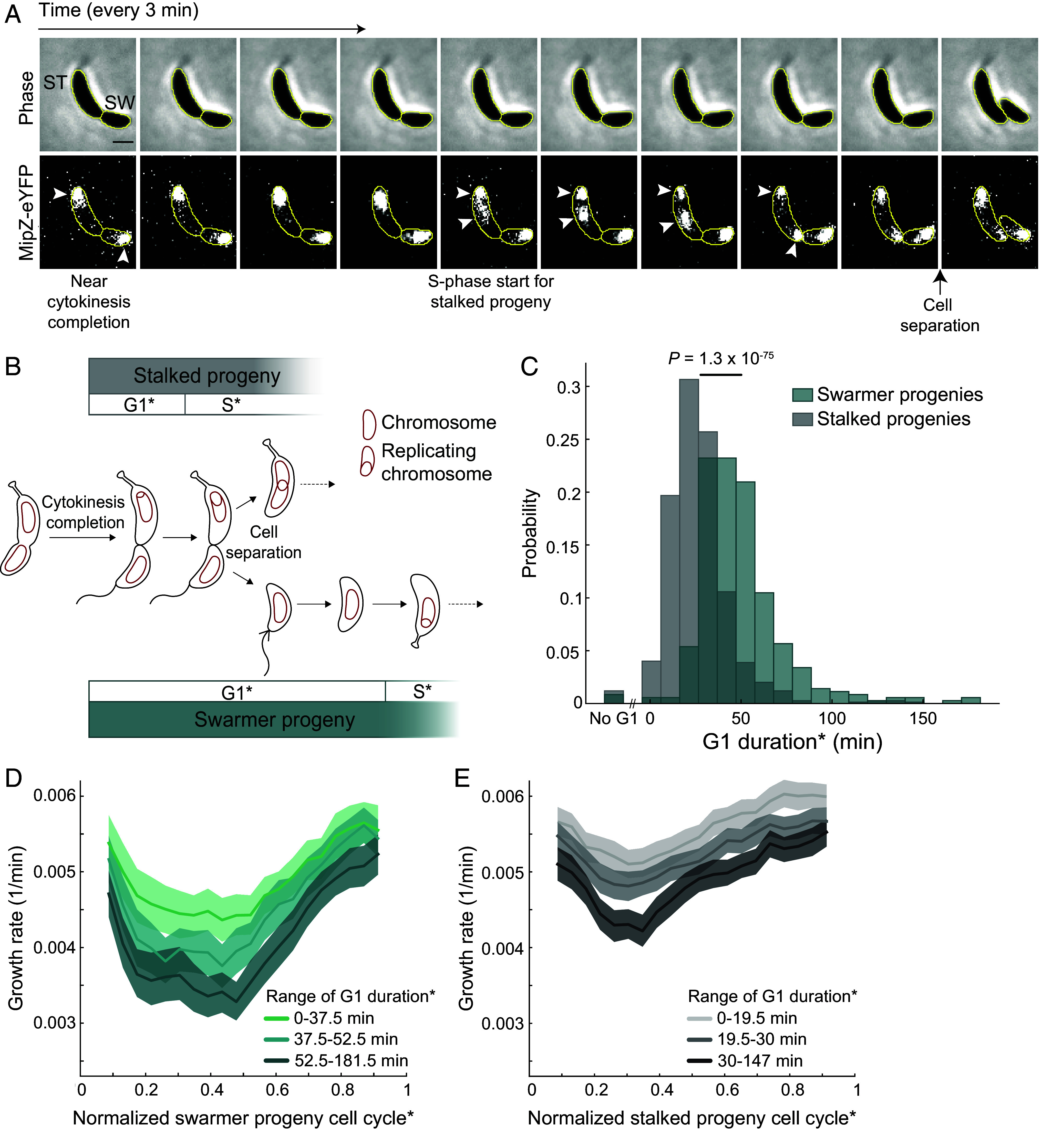
Cell segmentation of deeply constricted cells captures DNA replication of stalked progenies initiating in mother cells prior to daughter cell separation. (*A*) Example montage of a stalked progeny initiating DNA replication prior to cell separation. White arrowheads show when the MipZ-eYFP spot duplicates and segregates in the stalked cell compartment of the mother cell. Scale bar represents 1 µm. Cell contours indicate when the mother cell was segmented into two cells near the time of cytokinesis completion. (*B*) Schematic showing a revised representation of the start of G1 of *C. crescentus* progenies where cell birth is defined at the completion of cytokinesis in the mother cell, rather than cell separation. To identify measurements made under this revised definition, the cell cycle of each progeny and their associated cell cycle phases are marked by asterisks. Fade and arrows with dotted lines indicate cell cycle continues beyond schematic depiction. (*C*) Histogram of the absolute duration of the G1 phase* across all analyzed 353 swarmer and 747 stalked progenies under the new definition of cell cycle start. *P*-value is the result from the Mann–Whitney *U* test comparing the two distributions. (*D*) Plot showing the cell cycle growth rate of swarmer progenies binned by the time spent in the G1 phase*, which was from 0 to 37.5 min (n = 122 cells), 37.5 to 52.5 min (n = 119 cells), and 52.5 to 181.5 min (n = 112 cells). Solid lines and shaded areas denote mean and 95% CI of the mean from bootstrapping, respectively. (*E*) Same as panel (*D*) but for stalked progenies with G1 phase durations* of 0 to 19.5 min (n = 275 cells), 19.5 to 30 min (n = 251 cells), and 30 to 147 min (n = 221 cells).

Encouraged by these results, we built a computational pipeline based on the Omnipose/SuperSegger cell segmentation model and automated spot detection (*Materials and Methods*). This allowed us to reanalyze our time-lapse experiments under a different cell cycle definition in which progenies are born when the late predivisional mother cell is segmented into two cells (i.e., near the time of cytokinesis completion) instead of the time of visible daughter cell separation, as illustrated in Fig. 3*A*. Under this definition, the births of both progenies are effectively shifted to an earlier time, which occurs in the late predivisional stage of the mother cell (Fig. 3*B*). Hereafter, any cell cycle measurement that uses this definition will be labeled with an asterisk (e.g., G1 duration*, growth rate*, progeny cell cycle*) to distinguish from those that used cell separation to define the time of cell birth.

First, we verified that the average growth rate* of swarmer progenies remained lower than that of stalked progenies under our analysis pipeline and revised cell birth definition (*SI Appendix*, Fig. S2*D*). We also confirmed that our Omnipose/SuperSegger-based segmentation model was effective at capturing early events of DNA replication initiation in mother cells, as shown by the increased percentage of stalked progenies (98%, 735/747) with positive values of G1 durations* (Fig. 3*C* vs. Fig. 2*C*). We found that the growth rate slowdown started immediately (within the detection limit) for both progenies (Fig. 3 *D* and *E*). Note that the apparent truncation of the growth rate trajectories at the beginning and end of the swarmer and stalked cell cycles* is due to the data binning effect. Nevertheless, the immediate decrease in growth rate suggests that this event likely starts in the mother cell when cytokinesis is completed. The extent of the growth slowdown correlated with the G1 duration*, irrespective of daughter cell identity (Fig. 3 *D* and *E*).

Altogether, these results suggest the following points: i) the true G1 phase starts in the mother cell, likely at the time of cytokinesis completion prior to cell separation, ii) swarmer and stalked progenies are both subject to the G1-associated growth slowdown, but iii) swarmer progenies are more affected than their stalked siblings because their G1 phases are longer on average.

### SpoT Activity Accentuates the G1-Associated Cell Growth Slowdown.

Given that the severity of the growth slowdown correlated with the duration of the G1 phase, we hypothesized that molecular factors that affect this cell cycle phase may impact the growth rate profile during the cell cycle. Second messengers guanosine tetraphosphate and pentaphosphate [collectively referred to as (p)ppGpp] may be such factors, as their accumulation has been shown to lengthen the G1 phase of swarmer cells by indirectly modulating the abundance of two regulators of DNA replication initiation, DnaA and CtrA (44–48). Induction of (p)ppGpp production—either artificially or through nutrient starvation—delays the G1/S (or swarmer-to-stalked cell) transition (35, 45, 46). Under some nutrient-replete conditions, basal levels of (p)ppGpp can also affect cell physiology. For instance, loss of the bifunctional synthase/hydrolase enzyme SpoT, which abrogates (p)ppGpp production, lowers the fraction of G1 cells relative to the wild-type parent in steady-state populations growing in PYE (44). This suggests a shorter G1 phase when cells lack basal (p)ppGpp production. Interestingly, bulk culture measurements of the Δ*spoT* strain have also shown that this (p)ppGpp-devoid mutant has a slightly shorter doubling time in PYE medium relative to the wild-type strain (44). These observations motivated us to examine cell growth over the life cycle of the Δ*spoT* strain in PYE on low-agarose pads by timelapse imaging.

**Fig. 4.**
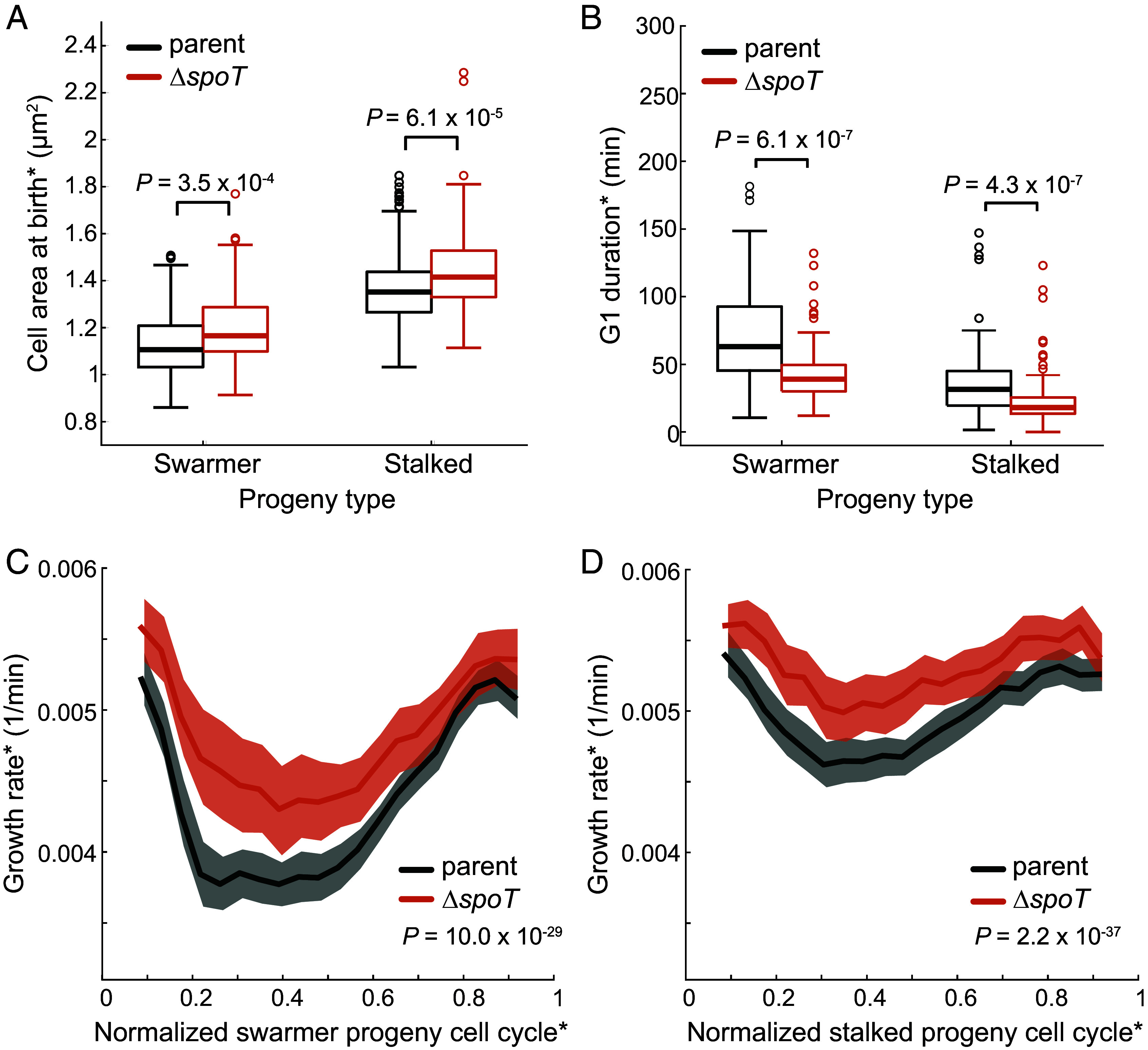
Loss of (p)ppGpp attenuates the G1 growth slowdown. (*A*) Box plot comparing cell area at birth* between wild-type parent (CB15N) and *ΔspoT* (CJW7364) strains for swarmer (n = 183 for wild-type, n = 86 for *ΔspoT*) and stalked (n = 211 for wild-type, n = 120 for *ΔspoT*) progenies. Thick central lines represent medians, box edges denote the interquartile range (from the 25th to the 75th percentile), whiskers indicate smallest and largest values within 1.5 times the interquartile range, and circles are single data points that fall outside of the interquartile range. *P*-values were obtained using the Mann–Whitney *U* test. (*B*) Same as (*A*) but for the G1 duration* between parent (CJW2022) and *ΔspoT* (CJW7511) strains for swarmer (n = 54 for parent, n = 90 for *ΔspoT*) and stalked (n = 83 for parent, n = 128 for *ΔspoT*) progenies. (*C*) Plot comparing the normalized growth rate of swarmer progenies between wild-type parent (CB15N, n = 183) and Δ*spoT* (CJW7364, n = 86) cells. Solid lines and shaded areas denote mean and 95% CI of the mean from bootstrapping, respectively. *P*-value was obtained using a two-sample Kolmogorov–Smirnov test. (*D*) Same as (*C*) but for the stalked progenies (n = 211 for parent and n = 120 for Δ*spoT*).

Quantitative image analysis of Δ*spoT* cells at birth revealed a small (5 to 7%) but statistically significant (Mann–Whitney *U* test, *P* ≤ 3.5 × 10^−4^) difference in average cell area relative to the wild-type cells imaged and segmented under the same conditions (Fig. 4*A*). This cell size difference was also observed for the strains carrying the MipZ-eYFP marker (*SI Appendix*, Fig. S3*A*). Swarmer and stalked progenies tended to have slightly faster average growth rates* when SpoT was absent (*SI Appendix*, Fig. S3*B*), suggesting that (p)ppGpp affects the growth rate of both cell types. Similarly, we found that the distribution of G1 duration* was shifted toward lower values in (p)ppGpp-devoid cells relative to the parent strain, irrespective of cell type (Fig. 4*B*). This suggests that (p)ppGpp delays the G1/S transition in not only swarmer, but also stalked progenies.

By extracting complete single-cell growth trajectories, we found that both swarmer and stalked Δ*spoT* progenies exhibited attenuated growth rate slowdowns compared to the wild-type parent strain (Fig. 4 *C* and *D*), consistent with our hypothesis. Similar effects were observed with the corresponding strains carrying the MipZ-eYFP marker (*SI Appendix*, Fig. S3 *C* and *D*). These results suggest that (p)ppGpp can accentuate the cell growth slowdown during the cell cycle.

## Discussion

While *C. crescentus* has been extensively studied with respect to asymmetric cell division, cell development, and intracellular organization for decades (16, 18, 49, 50), a characterization of cell growth over the complete cell cycle of both *C. crescentus* progenies has been missing. Here, we demonstrate that, under the experimental conditions tested, this bacterium modulates its growth in a manner coordinated with the G1 phase. Cell cycle-coordinated growth rate regulation is common in eukaryotes, though the pattern over the cell cycle can vary across cell types (28–30). From a broad perspective, the growth rate pattern of *C. crescentus* is reminiscent of that of some mammalian cells. Specifically, HeLa cells and retinal pigment epithelial cells exhibit a transient slowdown in growth rate during the G1 phase followed by an increase in growth rate at the S-phase transition (29). *C. crescentus* exhibits checkpoints and cell cycle controls typically not present in symmetrically dividing bacteria (10, 11, 16, 51, 52). As such, *C. crescentus* has long been considered to exhibit more eukaryote-like behavior. The cell cycle modulation of growth rate described here extends this metaphor into the realm of cell growth.

It is often assumed that the stalked daughter cell initiates DNA replication immediately after birth. This is in contrast to the swarmer daughter cell, which undergoes a protracted period of DNA replication arrest before entering S phase. While this view accurately reflects the average behavior of swarmer and stalked progenies, the picture is more nuanced at the single-cell level. We found that many stalked progenies initiate DNA replication in their mother cells and experience a period of DNA replication arrest during which their growth rates decrease (Fig. 3). Therefore, we propose to redefine the start of the G1 phase at the time of cytokinesis completion in the mother cell. This corresponds to the time when the future daughter cells already have separate cytoplasms, allowing for distinct biochemistries and differential activity of key cell cycle regulators such as CtrA and DivK (10, 39, 40). In effect, the G1 phase would become what we called G1 phase* in this study (Fig. 3*B*).

Notwithstanding these cell cycle definitions, it is clear that most swarmer progenies display considerably longer periods of DNA replication arrest than stalked progenies (Figs. 2 *C* and 3*C*), largely due to differences in CtrA activity between the two daughter cells (40). As a result, swarmer progenies are, on average, associated with a stronger growth slowdown during the cell cycle (Figs. 1*F* and 3 *D* and *E*), resulting in slower average growth rates relative to their stalked siblings (Fig. 1*B* and *SI Appendix*, Fig. S2*D*). This was observed not only in the complex PYE medium, but also in the defined medium M2G (*SI Appendix*, Fig. S4*A*). While we primarily used low (0.3%) agarose pads to image cells in this study, we found that tracking cells immobilized on conventional 1% agarose pads gave similar results in G1-associated growth slowdown and growth rate differential between daughter cells (*SI Appendix*, Fig. S4 *B* and *C*). Using low-agarose pads helped identify the time of cell separation through the release and motion of the swarmer daughter cell. This advantage becomes less important under the revised cell cycle definition in which cell birth and the G1 phase effectively start in the mother cell (Fig. 3). Conveniently, our image analysis pipeline performed well at splitting late predivisional cells, allowing the tracking of both swarmer and stalked progenies prior to their physical separation (*SI Appendix*, Fig. S2).

The observed growth slowdown suggests that *C. crescentus* does not use its maximal growth potential during the G1 phase. This raises the question of what this organism is prioritizing. In *C. crescentus*, the G1 phase of swarmer cells is associated with motility, chemotaxis, and surface sensing by polar organelles (53–57). The growth slowdown effectively extends the G1 lifetime and thus increases representation of G1-associated traits within the population, which may be ecologically beneficial by, for example, increasing species dispersal and exploration.

Given the functional importance of the G1 phase, the ability to further modulate its duration would seem an important factor in a cell’s ability to respond to a changing environment. Upon hard surface contact and mechanical stimulation, *C. crescentus* swarmer cells have been shown to shorten their G1 phase to more quickly differentiate into stalked cells and adapt to a surface-associated lifestyle (58). In our experiments, cells transitioned from liquid culture to growth on agarose pads. However, the surface of agarose pads is not hard enough to stimulate surface-contact responses (58, 59), which likely explains why cells grown on 1% agarose pads also displayed a growth slowdown (*SI Appendix*, Fig. S4*C*). It will therefore be interesting to examine the effect of harder surfaces on growth rate in future research.

Another environmental cue known to modulate the G1 duration is nutrient deprivation. Indeed, swarmer progenies are known to spend more time in the G1 phase upon nutrient limitation by accumulating (p)ppGpp (44, 46, 48, 60, 61). We show that the small level of (p)ppGpp present under the nutrient-replete condition of our timelapse experiments is sufficient to accentuate the G1 growth slowdown, presumably by extending the G1 phase (Fig. 4). This implies that higher accumulation of (p)ppGpp—as expected under carbon or nitrogen deprivation—should result in even more substantial growth slowdown. Consistent with this idea, artificial accumulation of (p)ppGpp in nonstarved cells is associated with an increase in doubling time at the culture level (45). A larger G1 growth slowdown through (p)ppGpp accumulation would effectively delay the costly process of DNA replication, which can account for a substantial fraction of the total energy used by the cell [~6% for *E. coli* in minimal growth medium (62)]. This could also help cells avoid toxic states, such as stalled DNA replication forks, if the conditions deteriorate to the point that DNA replication cannot be completed.

Asymmetric divisions appear common among α-proteobacteria despite their diversity of lifestyle and ecology (2–4, 6, 8, 13). In future studies, it will be valuable to examine whether a difference in growth rate between daughter cells is observed for other members of this class as well. Similar to what is observed in *C. crescentus*, the smaller daughter cells of the plant symbiont *Sinorhizobium meliloti*, the plant pathogen *Agrobacterium tumefaciens*, and the free-living species *Hyphomonas neptunium* initiate DNA replication later relative to their bigger siblings (13, 63, 64). The G1 phase has also been shown to be essential to the lifestyles of α-proteobacteria as a time during which cells acquire important traits associated with symbiosis, virulence, or dispersal (7, 8, 13). For instance, in the animal and human pathogen *Brucella abortus*, the G1-phase cell is the primary infectious form (7). Interestingly, synthesis of (p)ppGpp in *B. abortus* is required for proliferation in phagocytic cells and for survival in mice (65, 66). Conversely, overproduction of (p)ppGpp results in an accumulation of G1-phase cells in the population (67). In *Rhizobia* spp., mutants lacking (p)ppGpp are severely impacted in their ability to enter into a symbiotic relationship with the leguminous host plant (68, 69) whereas (p)ppGpp overproduction due to nitrogen deprivation delays the G1/S phase transition (70), drawing another parallel with *C. crescentus*.

While (p)ppGpp accentuates the cell growth slowdown during the cell cycle, it does not cause it because a transient decrease in growth rate still occurs in (p)ppGpp-devoid cells (Fig. 4 *C* and *D*). The mechanism underlying the growth slowdown remains unclear, though its evident connection to the G1 phase points to a potential role for G1 phase regulators (e.g., CtrA and DnaA). We hope that our findings will motivate future experimental and modeling studies that will examine cell growth modulation as another dimension to the fascinating life cycle of *C. crescentus*. By extension, it may ultimately become relevant to our understanding of pathogenesis, symbiosis, or other important processes associated with the G1 phase of other α-proteobacteria.

## Materials and Methods

### Bacterial Strains and Growth.

Bacterial strains and their constructions are listed in *SI Appendix*, Table S1. Phage transductions with ɸCR30 and conjugations with S17-1 strains were performed as described previously (71). The plasmid pNPTS138 was used for two-step gene replacement with counterselection, as described previously (72). Gibson assemblies were performed using the Gibson Assembly^®^ Master Mix from New England Biolabs (73). Primers are listed in *SI Appendix*, Table S2.

All strains were grown at 30 °C in PYE (2 g/L bacto-peptone, 1 g/L yeast extract, 1 mM MgSO_4_, 0.5 mM CaCl_2_) or M2G (0.87 g/L Na_2_HPO_4_, 0.54 g/L KH_2_PO_4_, 0.50 g/L NH_4_Cl, 0.2% (w/v) glucose, 0.5 mM MgSO_4_, 0.5 mM CaCl_2_, 0.01 mM FeSO_4_). To achieve steady-state growth, cells were grown in overnight (~18 h) cultures then diluted at least 1:10,000 in fresh medium and grown to exponential phase (OD_660_ < 0.3) in an incubator shaker (~300 rpm, New Brunswick Innova 44) prior to microscopy.

### Epifluorescence Microscopy.

Phase contrast and epifluorescence images were acquired on a Nikon Ti-E microscope equipped with a Perfect Focus System, a 100× Plan Apo λ 1.45 NA oil immersion objective, a motorized stage, and an Orca-Flash 4.0 V2 142 CMOS camera (Hamamatsu). For timelapse imaging, an objective heating ring was used set to 30 °C and phase images were collected every 1.5 min. When appropriate, YFP images were collected every 3 min. Chroma filter sets were used to acquire fluorescence images: yellow fluorescence protein (YFP, excitation ET500/20×, dichroic T515lp, emission ET535/30 m), cyan fluorescence protein (CFP, excitation ET436/20×, dichroic T455lp, emission ET480/40 m), and mCherry (excitation ET560/40×, dichroic T585lp, emission ET630/75 m). The microscope was controlled using Nikon software “NIS-Elements AR”. When performing timelapse imaging, cells were imaged on 0.3% agarose pads made with PYE or M2G unless indicated. For snapshot imaging, 1% agarose pads made with PYE were used.

### Image Processing and Analysis.

We use two methods to segment cells and determine the timing of cell birth. The first one uses visual inspection to determine the time when predivisional cells separate into swarmer and stalked progenies. Late predivisional cells were followed manually and birth was assigned as the frame before two daughter cells visually separated from each other. For this method, cell meshes were derived from cell segmentation of phase contrast images using the open-source image analysis software Oufti (74). Cell size characteristics (e.g., area and volume) were taken directly from Oufti or approximated using the MATLAB function Extract_Extended_Cell_Properties.m (75). Growth rate and associated cell characteristics were calculated using the MATLAB script cell_trajectory_analysis.m. In brief, cell area growth curves were smoothed over a 12-frame sliding-average window (equivalent to 18 min) and the difference in the cell area between consecutive frames was calculated. The relative growth rate was calculated by dividing the absolute growth rate by the cell area from the first of the consecutive frames. Residuals were calculated as the difference between the cell area trajectory and a first-degree polynomial fit to the log-transformed cell area trajectory for each cell. To approximate the timing of DNA replication initiation from timelapse images, the first frame at which the MipZ-eYFP cloud appeared expanded or separated into two distinct clouds (37) was manually assigned as the end of the G1 phase. G1-specific cell characteristics (e.g., G1 duration) were calculated using the MATLAB script cell_trajectory_analysis.m. This method was used for Figs. 1 and 2 and *SI Appendix*, Fig. S1.

The second method leveraged the Supersegger/Omnipose software (https://github.com/tlo-bot/supersegger-omnipose) for cell segmentation and used an Omnipose model that was retrained on *E. coli* cells for delaying the splitting of the cell masks during cell division (43). The Supersegger software (41) was used to correct the time-lapse data against drift and to link the cell masks over consecutive frames. The linked cell trajectories were then fed into a custom Python pipeline to perform the following steps: 1) To quantify cell features over time (import_data.py), single cell masks were cropped and phase contrast and fluorescence images were imported and processed to extract parameters such as cell area and lineage information for each cell. For MipZ-eYFP, DivK-eCFP, and DivK-mCherry foci detection, each cell was passed through a custom-made spot detection algorithm that identified the number of foci, their brightness, and their intracellular localization over time (spot_detection.py). 2) Poorly segmented cells and incorrectly linked cell trajectories were removed from further analysis (cell_curation.py). Exclusion criteria were based on trajectory length [i.e., too short (< 30 min) to represent true cell cycles], cell area increase during the cell cycle [shorter (< 0.61 μm^2^) than the size of any cell at birth], and instantaneous growth rates (where values > 0.015 min^−1^ and < −0.0025 min^−1^ were found to represent inaccurate cell segmentation upon visual inspection of the data). Additionally, only cells with both a mother and a daughter cell detected were retained for analysis. Consequently, only cells with a fully tracked cell cycle (i.e., birth to division) were further analyzed. 3) To classify stalked versus swarmer progenies (classify_progeny_type.py), curated cells were visually inspected one-by-one to determine their progeny type based on the presence of a stalk and/or cell motion. To allow for both quick and accurate assessment, zoomed-in and zoomed-out phase contrast images and masks of each cell from the beginning and end of their cell cycles were presented to the user for manual assignment of “Stalked” or “Swarmer” identity, or “Uncertain” when the identity could not be confidently ascribed. Stalked classification was assigned to cells that presented a visible stalk or whose sister cell was found to move considerably during the cell cycle. Conversely, Swarmer identity was assigned to motile cells that lacked a stalk. Cells that did not display any of these two scenarios were tagged as Uncertain and removed from analysis. This visual inspection also allowed for manual curation of bad cell masks, incorrect mother/daughter cell linkages, out-of-focus cells, and truncated cells by an image edge.

For timelapse experiments that used 1% agarose pads, cell identity was assigned only based on the clear presence of a stalk on one of the daughter cells given that the higher concentration of agarose prevented swarmer cells from moving. When the presence of a stalk was not evident, cells were cataloged as Uncertain and removed from analysis. 4) Features of classified cells were further processed to extract parameters of relevance (post_processing.py), such as interdivision time, average growth rate, instantaneous growth rate, cell area at birth, and division, etc. In the case of strains expressing MipZ-eYFP, the G1 phase was quantified by assessing factors such as duration, end time, and cell area when two MipZ-eYFP foci were detected. This quantification was based on the output of the spot detection algorithm (G1_quantification.py) and involved further curation to address poorly identified fluorescent foci caused by a low signal-to-noise ratio or proximity to other cells. For the Δ*spoT* versus parent comparisons (Fig. 4 and *SI Appendix*, Fig. S3), cells born on the agarose pad later than 60 min after the start of image acquisition were excluded from analysis to mitigate potential differences in adaptation to long-term growth on pads between the strains. For the analysis of DivK localization (*SI Appendix*, Fig. S2 *A*–*C*), a single cell was removed from the analysis because it showed aberrant unipolar localization of DivK at the swarmer pole (instead of the stalked cell pole) at an early stage of cell constriction.

The Python scripts used to run the analysis pipeline, the library file with the relevant custom-written functions (library.py), as well as the retrained Omnipose model used for cell segmentation (merge_model_omni.py) are available on GitHub (https://github.com/JacobsWagnerLab/published/tree/master/Glenn_et_al_2024) (76). This pipeline was used for Figs. 3 and 4 and *SI Appendix*, Figs. S2–S4.

### Quantification and Statistical Analysis.

All sample statistics and correlation coefficients were calculated using built-in MATLAB or Python functions. For the Mann–Whitney *U* tests, *P*-values were calculated using the built-in MATLAB function ranksum, or the mannwhitneyu function from the scipy.stats module of the SciPy Python library. For the Kolmogorov–Smirnov test, *P*-values were calculated using the ks_2samp function from the scipy.stats Python module. Spearman’s rank correlation coefficient was used to assess the degree of association between variables.

## Supplementary Material

Appendix 01 (PDF)

Movie S1. **Representative phase contrast timelapse movie of a wildtype stalked and swarmer progeny growing and dividing on a low-agarose pad**. Strain is wildtype (CB15N). Scale bar represents 2 μm.

Movie S2. **Video of low-agarose timelapse showing multiple wildtype cells growing and dividing**. Strain is wildtype (CB15N). Scale bar represents 2 μm.

Movie S3. **Representative timelapse movie of cells bearing MipZ-eYFP and growing on low-agarose pad**. Strain is CJW2022. Left panel is phase contrast, right panel is the YFP channel. Scale bar represents 2 μm.

## Data Availability

The dataset used in this study is available on BioStudies using the access code DOI: 10.6019/S-BIAD1327 (77). All original code developed as part of this study has been deposited in the publicly accessible GitHub code repository at https://github.com/JacobsWagnerLab/published/tree/master/Glenn_et_al_2024 (76).
