# Supplementary material for: Coupling of cell growth modulation to asymmetric division and cell cycle regulation in Caulobacter crescentus
Source: Proc Natl Acad Sci U S A. 2024 Oct 3;121(41):e2406397121. doi: 10.1073/pnas.2406397121 (PMC11474046; doi:10.1073/pnas.2406397121)
Supplement: Supplementary file 1 — Appendix 01 (PDF) [file pnas.2406397121.sapp.pdf]

Supporting Information for

Coupling of cell growth modulation to asymmetric division and cell cycle regulation in *Caulobacter crescentus*

Skye Glenn, Alessio Fragasso, Wei-Hsiang Lin, Alexandros Papagiannakis, Setsu Kato, and Christine Jacobs-Wagner

Correspondence to Christine Jacobs-Wagner
Email: jacobs-wagner@stanford.edu

This PDF file includes:

Figures S1 to S4
Legends for Movies S1 to S3
Tables S1 to S2
SI References

Other supporting materials for this manuscript include the following:

Movies S1 to S3

Supplemental Figures

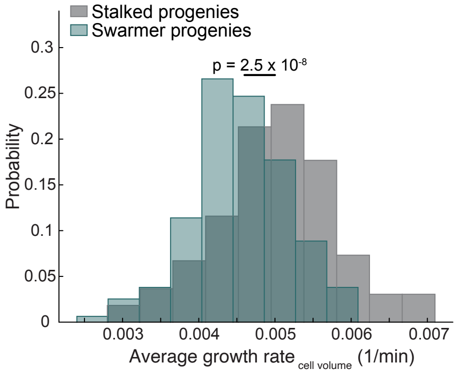

Fig. S1. Growth analysis of *C. crescentus* from low-agarose timelapse. Distributions showing the same cell size data as Fig. 1B but with average growth rate calculated from cell volume of stalked progenies (n = 164) and swarmer progenies (n = 158) of wildtype strain CB15N. The difference between the distributions between progeny types is statistically significant (Mann-Whitney U test, $p = 2.5 \times 10^{-8}$).

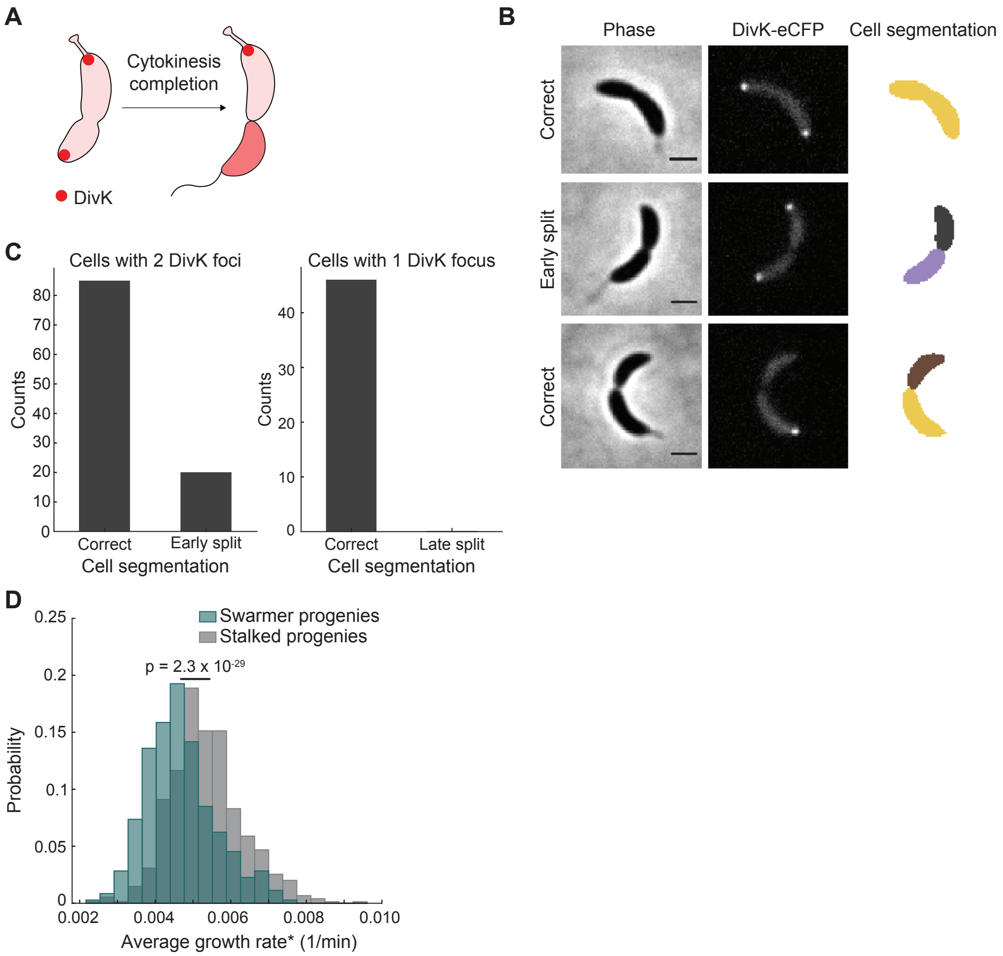

Fig. S2. The Omnipose/SuperSegger pipeline closely approximates timing of cytokinesis. (**A**) Schematic of DivK localization before and after cytokinesis completes in the late predivisional mother cell. (**B**) Images of CJW833 cells segmented by the Omnipose/SuperSegger cell segmentation model. Predivisional cells with two DivK-eCFP foci have a single contiguous cytoplasm and are, therefore, correctly identified as a single cell (top row), or split early and identified as two distinct cells (second row down), as denoted by two different colors in the "Cell segmentation" column. Only one DivK-eCFP focus located in the stalked compartment indicates a cell that has undergone cytokinesis. In this case, segmentation that identifies two cells (bottom row) is correct. Scale bars represent 1 μm. (**C**) Quantification of correct versus early or late cell segmentation mask splitting described in (B). (**D**) Histograms showing distribution of average growth rate of swarmer ($n = 353$) and stalked ($n = 747$) progenies calculated from cell segmentation and tracking using the Omnipose/Supersegger pipeline. Also shown is the p-value from a Mann-Whitney U test.

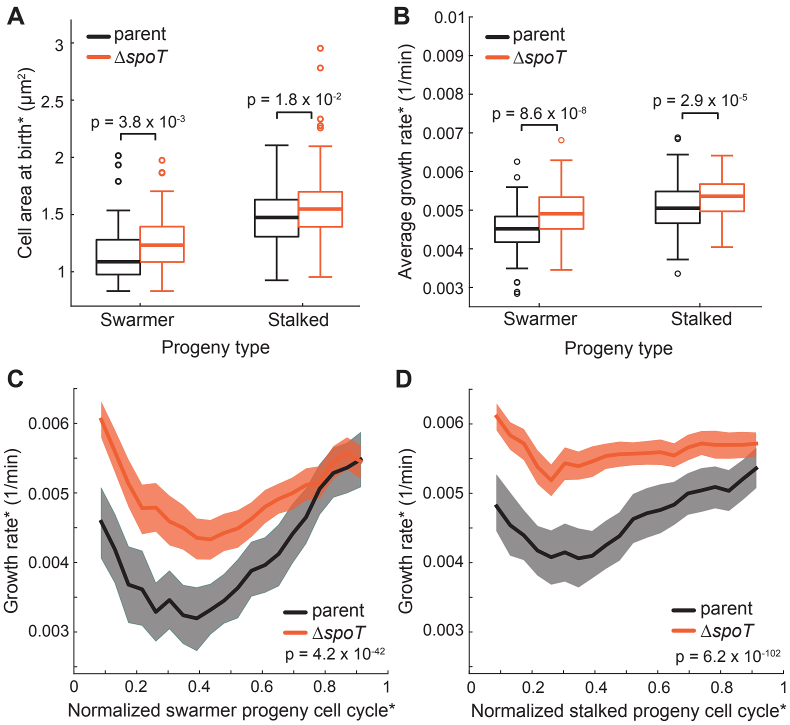

Fig. S3. Cells unable to produce (p)ppGpp exhibit attenuated G1 growth slowdown. (**A**) Box plot comparing cell area at cell birth* between parent (CJW2022) and Δ*spoT* (CJW7751) strains expressing *mipZ-eYFP* for swarmer (n = 54 for parent, n = 90 for Δ*spoT*) and stalked (n = 83 for parent, n = 128 for Δ*spoT*) progenies. Thick central lines represent medians, box edges denote the interquartile range (from the 25th to the 75th percentile), whiskers indicate lowest and highest values within 1.5 times the interquartile range, and circles are single data points that fall outside of the interquartile range. P-values are the results of Mann-Whitney U tests. (**B**) Box plot comparing the average growth rate* between wildtype parent (CB15N) and Δ*spoT* (CJW7364) strains for swarmer (n = 183 for wildtype, n = 86 for Δ*spoT*) and stalked (n = 211 for wildtype, n = 120 for Δ*spoT*) progenies. Plotting and statistical test are the same as panel (A). (**C**) Plot comparing the normalized growth rate of swarmer progenies between parent (n = 54) and Δ*spoT* (n = 90) cells expressing *mipZ-eYFP*. Solid lines and shaded areas denote mean and 95% confidence interval (CI) of the mean from bootstrapping, respectively. The p-value is the result of a two-sample Kolmogorov-Smirnov test. (**D**) Same as (C) except for stalked progenies (n = 83 for parent and n = 128 for Δ*spoT*).

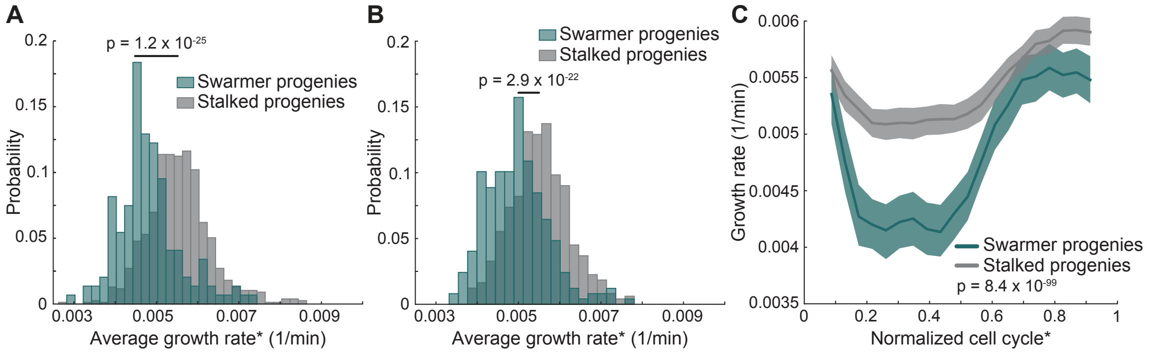

Fig. S4. Growth slowdown occurs in M2G medium and on 1% agarose pads. (**A**) Distributions of average growth rate for swarmer (n = 147) and stalked (n = 774) progenies of the wildtype strain CB15N growing in M2G on low-agarose pads. The p-value is the result of a Mann-Whitney U test. (**B**) Distributions of average growth rate for swarmer (n = 248) and stalked (n = 488) progenies of the *mipZ-eYFP* expressing strain (CJW2022) on conventional 1% agarose pads made with PYE. The p-value is the result of a Mann-Whitney U test. (**C**) Plot comparing the normalized growth rate of CJW2022 for swarmer (n = 248) and stalked (n = 488) progenies. Solid lines and shaded areas denote mean and 95% CI of the mean from bootstrapping, respectively. The p-value is the result of a two-sample Kolmogorov-Smirnov test.

Movie Legends

Movie S1 (separate file). Representative phase contrast timelapse movie of a wildtype stalked and swarmer progeny growing and dividing on a low-agarose pad. Strain is wildtype (CB15N). Scale bar represents 2 μm.

Movie S2 (separate file). Video of low-agarose timelapse showing multiple wildtype cells growing and dividing. Strain is wildtype (CB15N). Scale bar represents 2 μm.

Movie S3 (separate file). Representative timelapse movie of cells bearing MipZ-eYFP and growing on low-agarose pad. Strain is CJW2022. Left panel is phase contrast, right panel is the YFP channel. Scale bar represents 2 μm.

Tables

Table S1. *C. crescentus* strains used in this study.

Identifier	Genotype	Source
CB15N	parent strain	(1)
CJW833	CB15N *divK*::*divK-CFP*	(2)
CJW2022	CB15N *mipZ*::*mipZ-eYFP*	(3)
CJW7307	CB15N *divJ-CFP pleC-YFP divK*::*divK-mCherry*	A cross-over was conducted on strain CJW7336 using the *sacB* counterselectable marker to remove the plasmid sequence by plating on 3% sucrose. UV-inactivated ϕCR30 phage lysate was prepared from strain LS3205. *pleC-yfp* and *divJ-cfp* were transduced into the above and insertion was verified by fluorescence microscopy
CJW7336	CB15N *divK*::pNPTS138-divK-mCherry	pNPTS138 was amplified in using primers JSG_82 and JSG_83. The last 400 basepairs of *divK* were amplified out of CB15 using primers JSG_080 and JSG_081, and 400 basepairs C-terminal to *divK* using primers JSG_076 and JSG_077. mCherry was amplified from pCHYC-1 (4) using primers JSG_078 and JSG_079. The plasmid was assembled by Gibson assembly and electroporated into *E. coli* S17-1 cells. The S17-1 strain was conjugated with CB15N, followed by selection on kanamycin and nalidixic acid. Insertion was verified by fluorescence microscopy.
CJW7364	CB15N Δ*spoT*	pNPTS138 was amplified using primers JSG_104 and JSG_105. Approximately 1 kb of sequence immediately up- and down-stream of *spoT* was amplified from CB15N using primers JSG_106 with JSG_107, and JSG_108 with JSG_109, respectively. The plasmid was assembled by Gibson assembly and electroporated into *E. coli* S17-1 cells. The S17-1 strain was conjugated with CB15N, followed by selection on kanamycin and nalidixic acid. A second cross-over was conducted using the *sacB* counterselectable marker to remove the plasmid sequence. *spoT* deletion was confirmed by PCR amplification using the primers JSG_112 and JSG_113.
CJW7751	CB15N Δ*spoT mipZ*::*mipZ-eYFP*	pNPTS138 was amplified using primers JSG_104 and JSG_105. Approximately 1 kb of sequence immediately up- and down-stream of *spoT* was amplified from CB15N using primers JSG_106 with JSG_107, and JSG_108 with JSG_109,

		respectively. The plasmid was assembled by Gibson assembly and electroporated into *E. coli* S17-1 cells. The S17-1 strain was conjugated with CJW2022, followed by selection on kanamycin and nalidixic acid. A second cross-over was conducted using the *sacB* counterselectable marker to remove the plasmid sequence by plating on 3% sucrose. *spoT* deletion was confirmed by PCR amplification using the primers JSG_112 and JSG_113.

Table S2. Primers used in this study.

Identifier	Sequence (5' to 3')
JSG_076	AGTCCGTAATACGACTCACTTAAGGCCTTGACATGCGGTCGCCACGCCCG
JSG_077	CCACCGGCGGCATGGACGAGCTGTACAAGTAAAGCGCATCCGCGAGGGCG
JSG_078	GCCTCGCAACCGCCCTCGCGGATGCGCTTTACTTGTACAGCTCGTCCATG
JSG_079	AAAGGCAGCCTGCAGGCAGCGGCAGCGGCAGCGTGAGCAAGGGCGAGGAG
JSG_080	TCGCCCTTGCTCACGCTGCCGCTGCCGCTGCCTGCAGGCTGCCTTTCCAG
JSG_081	GGATGTACAGGCATGCGTCGACCCTCTAATGACGAAGAAGGTCCTCATCG
JSG_082	TATCCTCCACGATGAGGACCTTCTTCGTCATTAGAGGGTCGACGCATGCC
JSG_083	ACCGCGCTCGACGGGCGTGGCGACCGCATGTCAAGGCCTTAAGTGAGTCG
JSG_104	CGAGGACATCGTCACCACCGTAGCGAATTCGTGGATCCAGATATCCTGC
JSG_105	ACCACCTCCAGCCCGACCTGGCTTCGGCCGTGACGC
JSG_106	AGACGCGTCACGGCCGAAGCCAGGTCGGGCTGGAGG
JSG_107	AGGGTCCTCGTTCCGCCCCCCGCGCCCGATTCGGT
JSG_108	CGGGGACCGAATCGGGCGCGGGGGGCGGAACGAGG
JSG_109	CTGGATCCACGAATTCGCTACGGTGGTGACGATGTCCT
JSG_112	ACCTGGATCTCATCGCCCATG
JSG_113	ATGATCTTCTGCGCCAAGGCC

SI References

1. M. Evinger, N. Agabian, Envelope-associated nucleoid from Caulobacter crescentus stalked and swarmer cells. *J. Bacteriol.* **132**, 294–301 (1977).

2. H. Lam, J.-Y. Matroule, C. Jacobs-Wagner, The asymmetric spatial distribution of bacterial signal transduction proteins coordinates cell cycle events. *Dev. Cell* **5**, 149–159 (2003).

3. W. B. Schofield, H. C. Lim, C. Jacobs-Wagner, Cell cycle coordination and regulation of bacterial chromosome segregation dynamics by polarly localized proteins. *EMBO J.* **29**, 3068–3081 (2010).

4. M. Thanbichler, A. A. Iniesta, L. Shapiro, A comprehensive set of plasmids for vanillate- and xylose-inducible gene expression in Caulobacter crescentus. *Nucleic Acids Res.* **35**, e137 (2007).
